# Diagnostic Accuracy of Protein Glycation Sites in Long-Term Controlled Patients with Type 2 Diabetes Mellitus and Their Prognostic Potential for Early Diagnosis

**DOI:** 10.3390/ph11020038

**Published:** 2018-04-30

**Authors:** Sandro Spiller, Yichao Li, Matthias Blüher, Lonnie Welch, Ralf Hoffmann

**Affiliations:** 1 Institute of Bioanalytical Chemistry, Faculty of Chemistry and Mineralogy and Center for Biotechnology Biomedicine, Universität Leipzig, Deutscher Platz 5, 04103 Leipzig, Germany; 2 School of Electrical Engineering and Computer Science, Ohio University, Stocker Center 354, Athens 45701, OH, USA; yl079811@ohio.edu (Y.L.); welch@ohio.edu (L.W.); 3 Department of Medicine, Endocrinology and Nephrology, Universität Leipzig, Liebigstr. 21, 04103 Leipzig, Germany; Matthias.Blueher@medizin.uni-leipzig.de

**Keywords:** biomarker, fasting plasma glucose (FPG), glycated hemoglobin (HbA_1c_), glycation sites, multiple reaction monitoring (MRM), plasma proteins, type 2 diabetes mellitus (T2DM)

## Abstract

Current screening tests for type 2 diabetes mellitus (T2DM) identify less than 50% of undiagnosed T2DM patients and provide no information about how the disease will develop in prediabetic patients. Here, twenty-nine protein glycation sites were quantified after tryptic digestion of plasma samples at the peptide level using tandem mass spectrometry and isotope-labelled peptides as internal standard. The glycation degrees were determined in three groups, i.e., 48 patients with a duration of T2DM exceeding ten years, 48 non-diabetic individuals matched for gender, BMI, and age, and 20 prediabetic men. In long-term controlled diabetic patients, 27 glycated peptides were detected at significantly higher levels, providing moderate diagnostic accuracies (ACCs) from 61 to 79%, allowing a subgrouping of patients in three distinct clusters. Moreover, a feature set of one glycated peptides and six established clinical parameters provided an ACC of 95%. The same number of clusters was identified in prediabetic males (ACC of 95%) using a set of eight glycation sites (mostly from serum albumin). All patients present in one cluster showed progression of prediabetic state or advanced towards diabetes in the following five years. Overall, the studied glycation sites appear to be promising biomarkers for subgrouping prediabetic patients to estimate their risk for the development of T2DM.

## 1. Introduction

Diabetes mellitus (DM) is a group of diseases characterized by hyperglycemia resulting from absolute insulin deficiency (type 1 DM) or insulin resistance, together with a relative insulin secretion defect (type 2 DM). In 2013, DM affected approximately 382 million people worldwide andaccounted for more than 1.3 million deaths, making it the 8th leading cause of death and reduced life expectancy globally, according to a recent report of the World Health Organization (WHO) [1]. Around 90 to 95% of the currently estimated 415 million DM patients aged 20 to 79 suffer from type 2 DM (T2DM), which includes an estimated 193 million people who remain undiagnosed [2]. Already in the state of prediabetes, the risk for macrovascular complications is increased [3]. In this context, an early diagnosis of T2DM is urgently needed to increase the efficacy of established therapeutic strategies and to prevent or at least delay the development of complications, including macro- and microvascular diseases and diabetic neuropathy.

Recent data indicate that every third adult living in developed countries has prediabetes [4,5], which is an intermediate state of hyperglycemia defined as impaired fasting glucose (IFG) and/or impaired glucose tolerance (IGT). The latter is detected by an oral glucose tolerance test (OGTT). Both IFG and IGT are risk factors for type 2 diabetes, and risk is even greater when IFG and IGT occur together. Clinically, a prediabetic state is typically diagnosed (WHO criteria, [6]) by either fasting plasma glucose (FPG) levels between 6.1 and 7.0 mmol/L (110 and 125 mg/dL; IFG) or plasma glucose levels between 7.8 and 11.1 mmol/L (140 and 200 mg/dL) after a 2-h OGTT indicating IGT. The American Diabetes Association (ADA) uses the same IGT definition, but a reduced lower cut-off for IFG (5.6 mmol/L) and additionally glycated hemoglobin (HbA_1c_) levels between 5.7 and 6.4% as criteria [7]. Prediabetes is connected to a high risk for developing diabetes and associated complications [8] indicating that early detection of prediabetic states followed by immediate lifestyle interventions including weight reduction, calorie-reduced diets and increased physical activity will allow preventing or slowing down the transition from prediabetes to T2DM [9].

Current diagnostic criteria for the diagnosis of T2DM include plasma glucose levels above the borderline concentrations used for prediabetes, i.e., FPG ≥ 7.0 mmol/L (125 mg/dL) and OGTT ≥ 11.1 mmol/L (200 mg/dL), and HbA_1c_ levels ≥ 6.5% (48 mmol/mol) [10,11]. Worryingly, recent epidemiological studies indicate that HbA_1c_ and FPG tests with the currently applied diagnostic criteria only identify ~30–50% of previously undiagnosed T2DM patients [12,13,14,15,16]. In addition, HbA_1c_ poorly correlates with IFG and IGT [17,18,19]. Even the diagnosis of prediabetes based on IFG and IGT is still questionable due to the inability of these tests to predicti the development of the disease and the rather poor reproducibility in adults and children [20,21,22].

Besides HbA_1c_, a well-established diagnostic marker and glucose-control monitoring parameter determined in red blood cells, glycated serum proteins have been recognized as markers of hyperglycemia [23,24,25,26,27]. Due to the rapid turnover of serum proteins compared to erythrocytes (life span ~4 months), glycated proteins reflect mean concentrations of glucose in blood over a shorter period (days to weeks) than HbA_1c_ [28,29]. Thus, they might be useful biomarkers for monitoring short-term fluctuations of glucose plasma concentrations. Fructosamine and glycated albumin strongly correlate with HbA_1c_ and FPG [30,31,32,33,34], but the lack of clinical cut-off points and standardized assays are reasons for low clinical acceptance [29,35,36]. This might be attributed to the currently applied methods determining overall protein glycation degrees, whereas recent data indicate that specific glycation sites in plasma proteins might be more favorable biomarkers for early diagnoses of T2DM [37,38,39,40].

Recently, we reported 27 glycation sites of nine plasma proteins as markers of manifest T2DM [41]. All 27 glycation sites were present at significantly higher levels in samples from patients with T2DM compared to age- and body mass index (BMI)-matched control individuals. When combined with established diagnostic criteria, i.e., HbA_1c_ and FPG, the diagnostic accuracies improved significantly. Interestingly, glycation site Lys141 of haptoglobin (HP K141) provided the best sensitivities of ~94% and ~78% when combined with HbA_1c_ and FPG levels, respectively, and a specificity of ~98%.

Here, we extended our recent study by exploring the diagnostic value of 29 glycation sites originating from ten plasma proteins for prediabetes and long-term controlled T2DM patients. The glycation sites were quantified in 48 samples obtained from long-term controlled T2DM patients, 20 individuals with prediabetes, and 48 normoglycemic individuals.

## 2. Results

### 2.1. Long-Term Controlled Diabetic Patients

**Figure 1:**
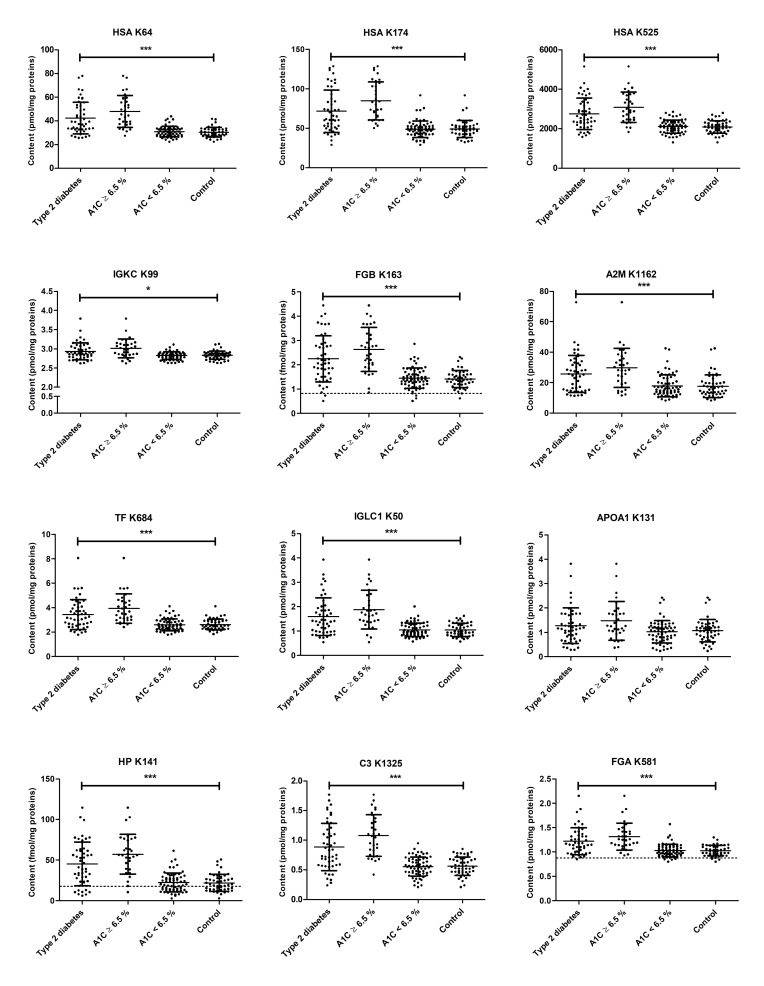
Contents of 12 glycated peptides in tryptic plasma digests obtained from long-term controlled type 2 diabetes mellitus (T2DM) patients and non-diabetic controls. Samples were split in two groups using an HbA_1C_ level of 6.5% as cut-off. Each dot represents the peptide level of the corresponding peptide in one plasma sample. Dotted lines indicate the limit of quantification (LOQ) of the peptide. Peptide sequences, glycation sites, and the corresponding protein are provided as Supplementary Materials (Table S2). Statistical significance was tested by a Mann-Whitney U-test (*** denotes *p* < 0.0001 and * denotes *p* < 0.05).

Twenty-seven out of the 29 analyzed glycated peptides were detected at significantly higher levels in digested sera from T2DM patients than in the control samples (*p* < 0.05, Figure 1), although both groups were not separated by a cut-off. When all T2DM and control samples were subdivided by an HbA_1c_ threshold of 6.5%, a similar distribution was obtained indicating that the glycation degrees of hemoglobin and the tested serum proteins correlated. Spearman’s rank correlation coefficients (*r_S_*), which were calculated for all glycated peptide levels and diagnostic parameters, ranged for most combinations of two glycated peptides from 0.37 to 0.98 (*p* < 0.001, data not shown). Moderate to strong correlations were achieved for all glycation sites with FPG (28 sites, 0.35 < *r_S_* <0.70, *p* < 0.001), proinsulin (20 sites, 0.37 < *r_S_* < 0.54, *p* < 0.001), HOMA-IR (23 sites, 0.35 < *r_S_* < 0.62, *p* < 0.001), and HbA_1c_ values for most sites (0.42 < *r_S_* < 0.76) (Table S11). Nine of the 14 glycation sites studied here for HSA and one site of fibrinogen beta chain (FGB K163) moderately correlated with free fatty acids (FFAs, 0.36 < *r_S_* < 0.46, *p* < 0.001), whereas correlations between peptide glycation and BMI were weak (−0.16 < *r_S_* < 0.25, 0.03 < *p* < 0.98).

Moderate to strong correlations were observed for HbA_1c_ values with HOMA-IR (*r_S_* = 0.73, *p* < 0.001) and FPG (*r_S_* = 0.79, *p* < 0.001) as well as alanine aminotransferase (ALAT), gamma glutamyl transferase (GGT), C-peptide, fasting plasma insulin (FPI), proinsulin, triglycerides (TAGs), and FFA (0.36 < *r_S_* < 0.68, *p* < 0.001). FPG moderately correlated (0.38 < *r_S_* < 0.68, *p* < 0.001) with aspartate aminotransferase (ASAT), ALAT, GGT, FPI, proinsulin, TAGs, HOMA-IR, and FFAs.

A ROC curve analysis relative to HbA_1c_ (cut-off 6.5%) and FPG (cut-off 7.0 mmol/L) provided for all glycated peptides maximal sensitivities (SNs) and specificities (SPs) up to 75% and 88%, respectively, and areas under curves (AUCs) up to 82% for certain cut-off concentrations (Table S12), which is in accordance with our recent data [42]. In comparison, sensitivities, specificities, and areas under curves of HbA_1c_ were 63%, 98%, and 89% (95% confidence interval (0.82–0.96)) and 54%, 98%, and 81% (95% confidence interval (0.72–0.91)) for FPG using the same cut-off values of 6.5% (likelihood ratio for positive results (LR+) = 29.8, likelihood ratio for negative results (LR−) = 0.38) and 7.0 mmol/L (LR+ = 25.8, LR− = 0.48), respectively. The analysis determined the best cut-off values maximizing sensitivities and specificities as 6.0% (LR+ = 19.5, LR− = 0.20) for HbA_1c_ (SN = 81% and SP = 96%) and 5.72 mmol/L (LR+ = 4.8, LR− = 0.25) for FPG (SN = 79% and SP = 83%). Interestingly, four further diagnostic parameters also showed sufficient values (AUC ≥ 80%) for the evaluation metrics: AUCs were 81% for C-peptide (cut-off 1.51 nmol/L; SN = 75%, SP = 77%), 84% for FPI (cut-off 115.0 pmol/L; SN = 81%, SP = 85%), 88% for HOMA-IR (cut-off 4.68, SN = 81%, SP = 94%), and 86% for HOMA2 %S (cut-off 36.6%, SN = 77%, SP = 98%).

As the diagnostic accuracies (ACCs) of all glycated peptides (61 to 79%) were insufficient, the data set was screened for variable combinations of each glycated peptide with HbA_1c_, FPG, C-peptide, FPI, HOMA-IR, and HOMA2 %S for optimal SN, SP, and ACC considering different cut-off points. However, only the combination with C-peptide showed a notably better ACC of 88% (Tables S5–S10).

**Figure 2:**
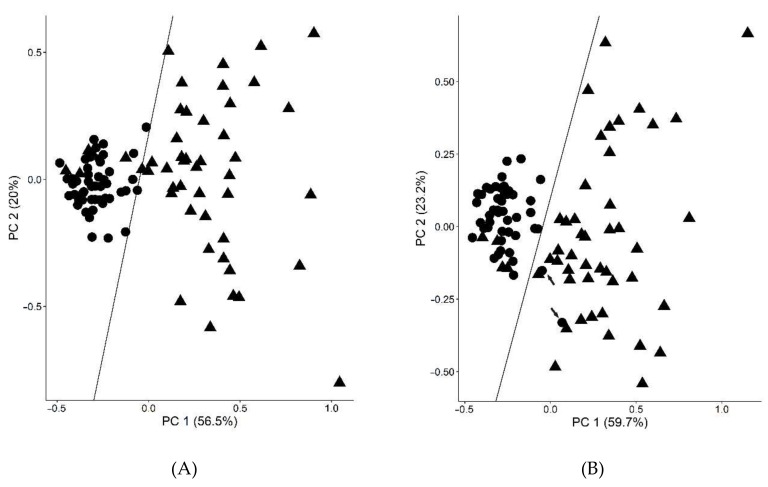
Principle component plots (PCP) of 48 T2DM patients (triangle) and non-diabetic controls (circle) using HbA_1C_, fasting plasma glucose (FPG), free fatty acids (FAA), C-peptide levels, HOMA-IR, and fasting plasma insulin (FPI) as diagnostic parameters with glycated peptide 26 (**A**) or without glycated peptide 26 (**B**) as seventh diagnostic parameter. False positives (**B**) are indicated by arrows reducing the SP from 96% to 92%. PC1 and PC2 denote first and second principle components, respectively.

**Figure 3:**
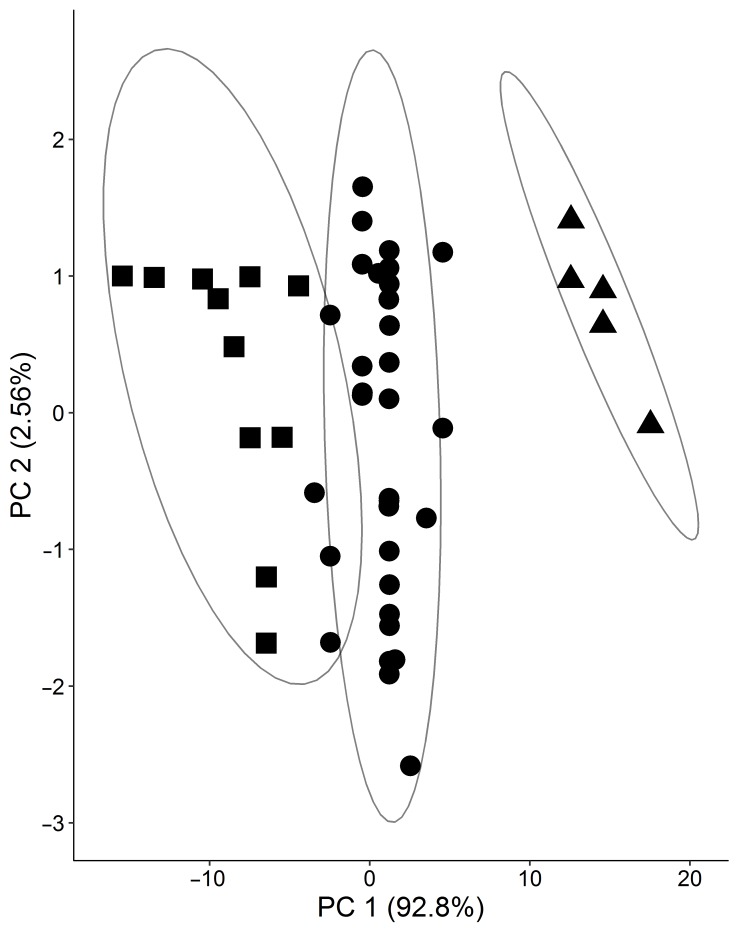
Principle component plot separating 48 T2DM patients in three clusters based on all available patient information, i.e., diagnostic parameters and glycated peptide levels. Missing values were imputed using Weka. The ellipses are drawn at the 95% confidence interval using the level parameter in the ggplot. PC1 and PC2 denote first and second principle components, respectively.

Thus, the RF-RFE method (random forest-recursive feature elimination) was applied to find a set of diagnostic parameters and glycated peptides for maximizing the classification of T2DM patients and controls. It revealed a set of seven features, i.e., C-peptide, FAAs, FPG, FPI, HbA_1c_, HOMA-IR, and glycated Lys141 of haptoglobin (HP K141, peptide 26), providing a SN of 94%, a SP of 96%, and an ACC of 95% (Figure 2A). To see the diagnostic contribution of peptide 26, a principle component analysis without the peptide was performed, showing two control samples (highlighted by arrows) being incorrectly classified as T2DM (Figure 2B). A cluster analysis performed for all 48 T2DM plasma samples using a k-means algorithm and considering all 29 glycated peptides and 43 clinical parameters identified three clusters as optimal considering the elbow criterion (Figure 3).

### 2.2. Prediabetic Patients

In addition to the diagnostic value of glycation sites, their potential as prognostic biomarkers was also investigated using samples of 20 males diagnosed with prediabetes. Peptide 19 (FGB K295, Supplement, Table S4) was removed from the dataset as it was always present at concentrations below its LODs, leaving 28 glycated peptides considered in the following statistical analyses. The correlation coefficients considering two glycated peptide levels typically ranged from 0.37 < *r_S_* < 0.99 (*p* < 0.05; data not shown). In combination with the other diagnostic parameters, correlations were usually weak (*r_S_* > 0.36, *r_S_* < −0.36), except for FFAs, FPI, and HOMA-IR that showed moderate correlation coefficients for selected peptides, i.e., between FFAs and 15 glycations sites (0.36 < *r_S_* < 0.57 (0.01 < *p* < 0.12), glycations sites Lys41, Lys75, and Lys99 of protein Ig kappa chain C region and FPI (*r_S_* = 0.63, 0.70, and 0.39, respectively; *p* < 0.05), and HOMA-IR (*r_S_* = 0.59, 0.67, and 0.40, respectively; *p* < 0.05). Moderate correlations were also observed for HbA_1c_ with total cholesterol (*r_S_* = −0.46, *p* = 0.04), HDL-cholesterol (*r_S_* = −0.56, *p* = 0.01), and OGTT (*r_S_* = 0.37, *p* = 0.1). Additionally, the levels of HDL-cholesterol and C-peptide were moderately correlated (*r_S_* = −0.37, *p* = 0.05). A cluster analysis of prediabetic plasma samples considering all glycation sites and a cluster stability test provided an optimal cluster number of three (Figure S1).

The recently reported cut-offs to classify newly diagnosed diabetic patients and control subjects [42] allowed subgrouping prediabetic men (Table S13) by counting how often each subject was above the cut-off values of all glycated peptide (Table S14). Intriguingly, three clusters could be distinguished again, i.e., 18–24 counts in cluster 1 (“highly-remarkable”), 6–12 counts in cluster 2 (“remarkable”), and up to 3 counts in cluster 3 (“unremarkable”). The clusters and the respective members were in agreement with the above-mentioned cluster analysis (Table S14 and Figure S1). Considering these three clusters, a RF-RFE method was applied to find a set of glycated peptides for maximizing the classification. A set of nine peptides representing eight glycation sites of HSA (Lys262, Lys378, Lys73, Lys525, Lys574, Lys359, Lys174, Lys64) and one of serotransferrin (Lys683) was identified, providing an ACC of 95% (Figure S2). Noteworthy, Lys262, followed by Lys378, Lys73, and K525 of HSA, contributed most to the classification verified by random forest feature importance (Table S15).

**Figure 4:**
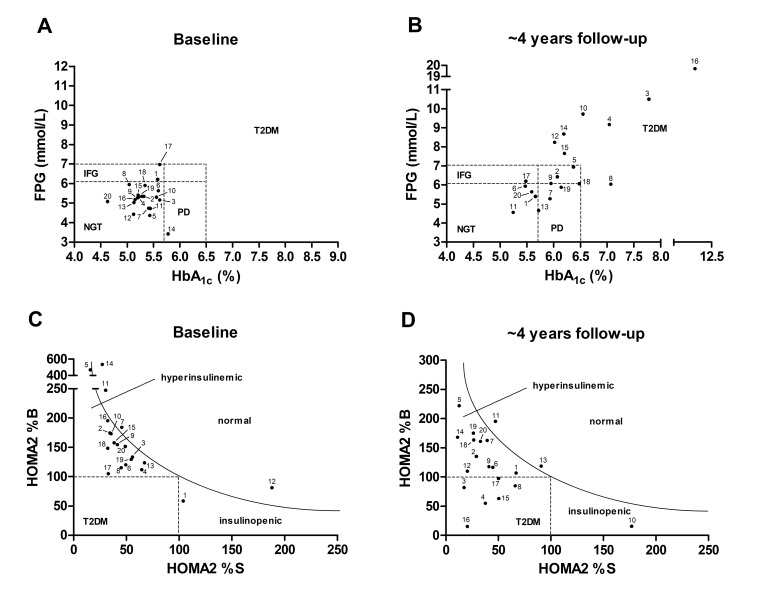
Categorization of twenty individuals with prediabetes (ADA criteria) by diagnostic parameters at baseline (**A**,**C**) and follow-up studies (**B**,**D**) using FPG versus HbA_1c_ levels (**A**,**B**) or HOMA2 %B versus insulin sensitivity HOMA2 %S values (**C**,**D**). Thresholds of FPG and HbA_1c_ levels (dashed lines) relied on the current diagnostic criteria of WHO and ADA for normal glucose tolerance (NGT), impaired fasting glucose (IFG), prediabetes (PD), and type 2 diabetes mellitus (T2DM). Updated homeostasis model assessment (HOMA2) separated individuals in normal, diabetic (100% values), insulinopenic or hyperinsulinemic state. The numbers indicate the prediabetes samples (Tables S14 and S15).

The predictive values of all glycation sites were evaluated by reexamining the individuals of clusters 1 to 3 after three to five years (Tables S2 and S3). Considering the diagnostic criteria of HbA_1c_ (>6.5%) and FPG (>7.0 mmol/L), eight persons converted from prediabetes to T2DM (ctT2DM), seven remained prediabetic with a clear trend towards diabetes (DPD, HbA_1c_ fold change: 1.10–1.21, FPG fold change: 0.93–1.59), and the glycemic status of five individuals remained stable or improved (PD, Figure 4A,B) within the 4 years observation period. Importantly, nine glycation sites, i.e., Lys93, Lys181, Lys262, Lys525, and Lys545 of HSA, Lys99 of Ig kappa chain C region, Lys1003 of alpha-2-macroglobulin, Lys50 of Ig lambda-1 chain C regions, and Lys120 of apolipoprotein A-I precursor were predominantly higher glycated in nine to twelve prediabetic patients. The nine glycation sites showed higher glycation degrees in six out of seven DPD plasma samples (86%), but only in four out of eight ctT2DM samples (50%).

Noteworthy, the status of all persons classified “remarkable” advanced towards diabetes with four already diagnosed with T2DM. Among the six patients classified as “highly remarkable”, only one advanced to T2DM, while half of the “unremarkable” group developed T2DM. In addition, highest HOMA-IR values (>10) and the strongest HOMA-IR changes (>5.2 to 16.3) were observed for persons classified “remarkable” (Supplement, Table S2). In general, HOMA2 %B (*n* = 14; 3–92% loss) and HOMA2 %S values (*n* = 13, 10–89% loss) decreased in most persons, which were diagnosed with hyperinsulinemia or diabetes (Figure 4C,D). Noteworthy, recommendations for healthy diet and increased physical activity were followed by the patients with a low (<50%) adherence rate (BMI fold change: 0.97 ± 0.05) and therefore, this intervention could not prevent progression of the prediabetic state or development towards T2DM.

## 3. Discussion

Recently, we quantified glycation sites in plasma proteins that might be valuable diagnostic markers to complement currently established diagnostic criteria based on HbA_1c_ and FPG [42], as both criteria failed to detect T2DM, especially in early phases. In this study, persons newly diagnosed with T2DM using different criteria (HbA_1c_, FPG, OGTT, or random plasma glucose) showed characteristic glycation patterns in plasma proteins that allowed their differentiation from matched healthy controls. The highest sensitivity to diagnose chronic hyperglycemia could be achieved by a combination of the glycation levels of four sites in plasma proteins, i.e., K93, K262, and K414 in HSA and K141 in haptoglobin, in combination with other routine parameters including HbA_1c_. This feature set provided a favorable diagnostic accuracy of around 98% compared to only 76 and 70% when only HbA_1c_ and FPG, respectively, were used. These promising results motivated us to extend our previous studies by including individuals with varying degrees of disturbances of glucose metabolism and insulin sensitivity who have been monitored for ~4 years. Here, we investigated the diagnostic and prognostic potential of plasma protein glycation sites longitudinally. Thus, we quantified 29 glycation sites originating from ten proteins with different half-life times in blood (e.g., 2 to 4 days for haptoglobin and 20 d for albumin) [35,43], in plasma samples from long-term controlled diabetic patients (*n* = 48) and matched non-diabetic subjects (control, n = 48). In addition, we included analyses of plasma samples obtained from 20 individuals who have initially been classified as prediabetic and re-evaluated after 3–5 years.

A ROC analysis of the data set clearly indicated that the glycation levels of the 29 investigated sites typically provide better SNs (up to 79%) than both HbA_1c_ (SN = 63%, AUC = 89%) and FPG (SN = 54%, AUC = 89%) and only slightly lower AUCs (up to 82%) for classifying T2DM patients and controls. Noteworthy, the three diagnostic parameters FPI (cut-off 115.0 pmol/L; AUC = 84%), HOMA-IR (cut-off 4.68, AUC = 88%), and HOMA2 %S (cut-off 4.68, AUC = 86%), which are typically used to characterize insulin resistance, provided clinically relevant AUCs. Additionally, data reiterate that lowering the cut-off values of HbA_1c_ (from 6.5 to 6.0%) and FPG (from 7.0 to 5.72 mmol/L), as recommended by the WHO, improved the SNs from 63 to 81% and from 54 to 79%, respectively, supporting recent reports [12,13,14,15,16].

In contrast to our previous study on newly diagnosed T2DM patients [42], glycation degrees of long-term controlled patients showed moderate to strong correlations with HbA_1c_ and other parameters of glucose metabolism and insulin sensitivity including FPG and HOMA-IR. This indicates that plasma proteins follow similar glycation kinetics as hemoglobin after manifestation of the disease and that their glycation degree generally increase as insulin sensitivity deteriorates. The previously reported negative correlation of BMI with glycation sites (57) was not observed in the current cohort, suggesting the assumption that the higher capacity of adipose tissue in patients with obesity leads to higher uptake of excess glucose is not valid after manifestation of T2DM.

Statistical evaluation of the data revealed a feature set matrix using one glycation site of haptoglobin (K141) and twelve routine parameters typically used to characterize T2DM (FPG, HbA_1c_, FPI, C-peptide) and IR (HOMA-IR, FFAs) [44], providing a high accuracy of 95% for the cohort, which confirms our previous results [42]. This result emphasizes the relevance of HP K141 acting as biomarker for the disease, as HP K141 also provided the highest accuracies in combination with HbA_1c_ and FPG in the previous cohort (57).

Based on cut-off values for the 29 glycation sites determined previously in patients with newly diagnosed T2DM [42], prediabetic subjects could be subdivided into three clusters. Importantly, these three clusters may reflect the individual risk to progress from prediabetes to T2DM, to remain prediabetic or to improve hyperglycemia. Reexamination of these individuals after three to five years indicated that individuals of one cluster predominantly advanced to T2DM or deterioration of prediabetic status. According to the RF-RFE method, glycation sites of HSA and serotransferrin contributed most to the classification of the three clusters at baseline, underlining their relevance for identifying prediabetic subgroups. Since subgroups might respond differently to T2DM treatment strategies or show individual risks for disease progression and developing diabetic complications, the glycation sites might provide a prognostic tool to overcome current diagnostic limitations. However, this study was limited by the small size of discovery cohorts and the limited transferability into clinical routine, providing ‘only’ first hints and the need to be tested in larger cohorts using at least the set of glycation sites applied here, maybe even further sites from different plasma proteins. Moreover, the value of our glycation site cluster to predict T2DM should be tested in the context of large, prospective epidemiological studies on the long-term incident risk to develop T2DM. In addition, we propose to investigate whether the combination of HbA_1c_ with glycation levels of plasma proteins (especially HSA, serotransferrin, and haptoglobin) and their dynamics may reflect the individual risk to develop long-term complications of diabetes including diabetic retinopathy, nephropathy and neuropathy.

## 4. Materials and Methods

### 4.1. Study Participants

Forty-eight patients with a duration of T2DM for >10 years and 48 non-diabetic individuals matched for gender, BMI, and age (range: 20–70 years) as well as 20 prediabetic men (age range: 25–60 years) were included in this study (Tables S1–S3). Anthropometric and laboratory chemistry parameters were measured for all plasma samples or calculated as previously described [45,46]. The study was approved by the Ethics Committee of Universität Leipzig (approval no: 159-12-21052012) and performed in accordance to the declaration of Helsinki. All subjects gave written informed consent before taking part in this study. T2DM and prediabetes were diagnosed according to the criteria of ADA [47]. T2DM patient samples were grouped by HbA_1c_ levels, i.e., <6.5% (male *n* = 7, female *n* = 11) ≥6.5% (male *n* = 17, female *n* = 13). Patients with HbA_1c_ < 6.5% (48 mmol/mol) were diagnosed on the basis of repeated FPG (>7.0 mmol/L) or OGTT (>11.1 mmol/L) assessments [47]. Some individuals of the T2DM group received anti-hyperglycemic medication (metformin, DPP-4 inhibitors). Prediabetic individuals (*n* = 20, BMI ≥ 30 kg/m^2^, age at baseline: 30–61 years) were identified according to ADA criteria [47]. After a mean observation period of ~4.1 years, blood samples were taken again from prediabetic subjects for a follow-up examination to measure the same clinical parameters as previously. All subjects had a BMI ≥ 25.0 kg/m^2^ and were therefore included into a multimodal intervention program consisting of regular dietary advice and 1-2 times per week physical exercise. Noteworthy, the adherence rate to this program was <50%. EDTA blood samples were collected after a twelve-hour fasting period between 8 am and 9 am, centrifuged (500× *g*, 5 min), and an aliquot was used to determine routine laboratory parameters within one hour. Cell debris was removed from the remaining aliquot by filtration (Rotilabo^®^ syringe filter, Carl Roth GmbH + Co. KG, Karlsruhe, Germany) and stored at –80 °C for analysis of the glycation sites. Plasma insulin and proinsulin were measured with an enzyme immunometric assay (IMMULITE automated analyzer, Diagnostic Products Corporation, Los Angeles, CA, USA). Serum high-sensitive CRP (C-reactive protein) was measured by immunonephelometry (Dade-Behring, Milan, Italy). HbA_1c_, plasma glucose, serum total high-density lipoprotein (HDL) cholesterol, low-density lipoprotein (LDL) cholesterol, triglycerides, and free fatty acids were measured as previously described [45]. Homeostasis model assessment as an index of insulin resistance (HOMA-IR) was calculated by multiplying the FPG (mmol/L) with fasting plasma insulin (FPI, mU/L) divided by 22.5 [48]. Updated HOMA (HOMA2) of insulin sensitivity (HOMA2 %S) and an estimate of beta cell function (HOMA2 %B) were calculated using FPG and FPI (HOMA calculator v2.2.3 at http://www.dtu.ox.ac.uk/homacalculator) [49].

### 4.2. Peptide Quantification

Twenty-nine glycation sites were quantified at the peptide level (Table S4) by electrospray ionization mass spectrometry (ESI-MS) on a QTRAP4000 (AB Sciex, Darmstadt, Germany) coupled on-line to reversed-phase high-performance liquid chromatography (RP-HPLC) using timed multiple reaction monitoring (MRM) [41]. Briefly, plasma was ultrafiltrated (5 kDa cut-off), digested with trypsin (37 °C, 18 h, 5% *w*/*w*), spiked with a concentration-balanced mixture of 13C,15N-labelled glycated peptides as internal standard, enriched for glycated peptides by boronic acid affinity chromatography (BAC), and desalted by solid phase extraction (SPE) [38,50,51]. Peptides were separated on a C18-column (AdvanceBio Peptide Mapping column, pore size 12 nm, length 15 cm, internal diameter 2.1 mm, particle size 2.7 µm, Agilent Technologies, Böblingen, Germany) coupled on-line to the QTRAP4000. Eluents A and B were water and acetonitrile, respectively, containing both formic acid (0.1%, *v*/*v*). The column was equilibrated with 3% eluent B, the sample injected, and peptides were eluted by linear gradients starting 3 min after sample injection to 10% eluent B within 1 min, to 20% eluent B within 10 min, and finally to 95% eluent B in 7 min. The flow rate was 0.3 mL/min and the column temperature was set to 60 °C. Quantification relied on timed MRM using specific transitions of each targeted and isotope-labelled peptide by integrating individual peaks in extracted ion chromatograms (XICs) using Analyst 1.6 software (AB Sciex) [41]. The quantities of all twenty-nine glycated peptides were normalized to the total protein content of each plasma sample determined by a Bradford assay [32]. Briefly, Coomassie Brilliant Blue G-250 solution (250 µL, 0.1 g/L in 10% H_3_PO_4_ in 5% aqueous ethanol) was mixed with the sample (5 µL) in duplicates in a 96-well microtiter plate and the absorbance recorded at 595 nm. Quantification relied on a 2-fold dilution series of bovine serum albumin (BSA; 1.0 mg/L to 62.5 µg/L).

### 4.3. Statistics and Bioinformatics

Datasets were evaluated by statistical tests, i.e., Kolmogorow-Smirnow, Mann-Whitney, and *t*-test, and Spearman rank correlation coefficients using Prism 6 (GraphPad software; La Jolla, CA, USA). Receiver operating characteristic (ROC) analyses and screening for variable combinations relied on the Excel-add-in Multibase 2015 (Numerical Dynamics, Tokyo, Japan) and Prism 6 software, respectively.

Diabetes and control samples were classified by a decision tree algorithm using HbA_1c_, FPG, C-peptide, FPI, HOMA-IR, and HOMA2 %S (Tables S5–S10) in combination with each glycated peptide. The decision tree algorithm was implemented using Scikit-Learn [52]. Accuracies were evaluated using nested 10-fold cross validation [53]. The best feature set for classification was identified by a RF-RFE method [54] that was applied for all glycated peptides and clinical parameters, such as HbA_1c_, FPG, and BMI. Feature normalization and missing value imputation relied on the WEKA toolkit [55]. Accuracies were evaluated using nested 10-fold cross validation [53]. The k-means algorithm in Scikit-Learn [52] was applied to find subclasses in diabetic samples. The clustering stability score [56] and elbow criterion [57] were used to find the optimal number of subclass. Positive (+) and negative (−) likelihood ratios (LR) were calculated as previously reported [58,59].

## 5. Conclusions

The data obtained here for small, well defined cohorts of long-term diabetic and prediabetic patients confirms the diagnostic potential and for the first time indicates the prognostic value of glycation sites of plasma proteins, which provide similar or better diagnostic accuracies as routinely applied clinical parameters. Interestingly, the combination of glycation sites and established clinical parameters provided the best accuracy (95%). Moreover, the studied glycation sites can subgroup prediabetic patients, allowing an estimation of the individual risk of patients to develop T2DM in the following years, which identify persons subject to early therapeutic inventions beyond dietary changes and exercises. In all cases, certain glycation sites of serum albumin, serotransferrin, and haptoglobin provided the best diagnostic and prognostic measures.

## Supplementary Materials

The following are available online at http://www.mdpi.com/1424-8247/11/2/38/s1.
