# Supplementary material for: Diagnostic Accuracy of Protein Glycation Sites in Long-Term Controlled Patients with Type 2 Diabetes Mellitus and Their Prognostic Potential for Early Diagnosis
Source: Pharmaceuticals (Basel). 2018 Apr 30;11(2):38. doi: 10.3390/ph11020038 (PMC6027301; doi:10.3390/ph11020038)
Supplement: Supplementary file 1 [file pharmaceuticals-11-00038-s001.pdf]

Diagnostic accuracy of protein glycation sites in long-term controlled type 2 diabetes mellitus and the prognostic potential for early diagnosis

Sandro Spiller, Yichao Li, Matthias Blüher, Lonnie Welch, Ralf Hoffmann

Electronic Supplementary Information

Directory

Table S1 2
Table S2 3
Table S3 4
Table S4 5
Table S5 6
Table S6 7
Table S7 8
Table S8 9
Table S9 10
Table S10 11
Table S11 12
Table S12 13
Table S13 14
Table S14 15
Table S15 16
Figure S1 17
Figure S2 18

Table S1. Characterization of T2DM patients, non-diabetic and prediabetic persons enrolled in this study.

	Control group		Prediabetic status	Diabetes patients (HbA_{1c} < 6.5%)		Diabetes patients ($HbA_{1c} \geq$ 6.5%)	
Variable	male	female	male	male	female	male	female
n	24	24	20	7	11	17	13
Age (years)	48 ± 8	53 ± 13	47 ± 9	52 ± 9	56 ± 7	47 ± 8	54 ± 15
Height (cm)	177 ± 9	165 ± 7	182 ± 6	183 ± 5	166 ± 4	179 ± 10	168 ± 7
Body weight (kg)	133 ± 26	101 ± 12	126 ± 23	129 ± 19	102 ± 16	144 ± 35	107 ± 16
BMI (kg/m^2)	42.6 ± 7.6	37.1 ± 4.3	37.6 ± 5.5	38.6 ± 5.7	37.0 ± 4.7	44.4 ± 7.7	38.5 ± 3.4
HbA_{1C} (%)	5.4 ± 0.5	5.4 ± 0.4	5.3 ± 0.3	5.6 ± 0.8	5.8 ± 0.5	8.2 ± 1.4	7.7 ± 1.2
FPG (mmol/L)	5.4 ± 0.7	5.3 ± 0.6	5.2 ± 0.7	5.5 ± 1.1	6.1 ± 1.4	9.0 ± 3.2	9.9 ± 3.8

Data are means ± SD for the subjects of each group. BMI - body mass index, FPG - fasting plasma glucose, HbA_{1c} - glycated hemoglobin.

Table S2. Disease relevant parameters of prediabetic subjects at baseline and follow-up studies.

Subject #[*]	Treatment[†]	Age at baseline	Duration follow-up examination [a]	BMI (kg/m^2)		HbA_{1c} (%)		FPG (mmol/L)		FPI (pmol/L)		HOMA1-IR		HOMA2 %B (%)		HOMA2 %S (%)	
				baseline	follow-up	baseline	follow-up	baseline	follow-up	baseline	follow-up	baseline	follow-up	baseline	follow-up	baseline	follow-up
6	d/e	47	4	40.5	39.9	5.59	5.47	5.64	5.93	106.5	116.9	3.8	4.4	120.1	116.3	49.4	44.6
7	no	44	4	35.4	36.3	5.41	5.93	4.74	5.28	121.0	137.5	3.7	4.6	183.7	162.6	45.6	39.2
9	d/e	56	4	41.7	40.7	5.22	5.95	5.31	6.07	129.8	127.1	4.4	4.9	154.6	117.9	41.4	40.9
13	d/e	54	5	37.1	33.6	5.13	5.72	5.03	4.66	80.0	60.1	2.6	1.8	123.5	118.5	67.2	90.8
18	no	48	3	32.2	33.0	5.34	6.48	5.90	6.06	163.5	201.3	6.2	7.8	148.4	163.3	32.3	26.4
20	no	61	3	33.4	34.0	4.63	5.59	5.07	5.64	110.4	162.6	3.6	5.9	151.4	160.9	49.0	32.9
2	no	43	5	35.2	36.4	5.32	6.07	5.34	6.42	156.9	179.4	5.4	7.4	174.2	135.4	34.4	29.0
3	d/e	45	5	48.2	49.0	5.62	7.78	5.16	10.50	96.3	277.7	3.2	18.7	133.3	82.0	55.7	17.3
5	no	28	3	45.1	50.5	5.43	6.37	4.37	6.94	384.4	431.4	10.8	19.2	467	222.1	15.7	12.6
10	no	56	5	38.1	38.3	5.56	6.55	5.30	9.72	152.2	25.9	5.2	1.6	173.1	15.5	35.5	176.8
12	no	30	4	31.7	32.2	5.12	6.02	4.43	8.24	29.2	246.8	0.8	13.0	81.0	109.9	187.9	20.3
14	no	33	5	48.9	50.0	5.78	6.19	3.42	8.67	230.8	475.0	5.1	26.4	535.2	168.1	27.1	11.1
1	no	56	3	32.1	33.1	5.58	5.66	6.21	5.39	48.8	79.1	1.9	2.7	58.1	107	103.8	66.8
4	no	47	4	33.4	33.5	5.28	7.04	5.34	9.17	82.2	126.6	2.8	7.4	111.8	55.1	64.5	37.6
8	no	53	5	36.3	36.8	5.04	7.07	5.94	6.04	115.9	78.0	4.4	3.0	115.3	84.8	45.0	65.9
11	no	34	5	32.5	32.8	5.44	5.24	4.72	4.56	185.4	118.4	5.6	3.5	247.3	195.3	30.3	47.1
15	d/e	52	3	34.0	30.5	5.21	6.2	5.40	7.65	139.8	97.5	4.8	4.8	157.5	63.2	38.4	50.3
16	d/e	52	4	40.2	40.9	5.15	12.06	5.17	19.70	169.4	114	5.6	14.4	195.3	15.4	32.3	20.4
17	no	49	4	38.8	36.5	5.62	5.48	6.97	6.20	154.7	102.9	6.9	4.1	104.7	97.7	32.8	50.0
19	d/e	53	4	41.2	39.1	5.20	6.14	5.27	5.87	97.9	204.5	3.3	7.7	129.3	175.2	54.5	26.2

[*] Numbering of persons refers to Table S6. Color code identifies persons as part of cluster 1 (red, “highly-remarkable”), cluster 2 (orange, “remarkable”), and cluster 3 (green, “unremarkable”) (for details see Table S7). † Subjects received no treatment (no) or changed their diet and did physical exercises (d/e). BMI - body mass index, FPG - fasting plasma glucose, HbA_{1c} - glycated hemoglobin, FPI – fasting plasma insulin, HOMA1-IR - homeostasis model assessment as an index of insulin resistance, HOMA2 %S - updated HOMA (HOMA2) of insulin sensitivity, HOMA2 %B - HOMA2 of beta cell function.

Table S3. State of prediabetic persons according to established diagnostic parameters, i.e., HbA_{1c} and FPG, at baseline and follow-up.

Subject #*	Treatment†	State at baseline	State at follow-up	BMI fold change	HbA_{1c} fold change	FPG fold change	FPI fold change	HOMA-IR fold change	HOMA2 %B fold change	HOMA2 %S fold change
6	d/e	NGT	NGT	0.99	0.98	1.05	1.10	1.16	0.97	0.90
7	no	NGT	PD	1.03	1.10	1.11	1.14	1.24	0.89	0.86
9	d/e	NGT	PD	0.98	1.14	1.14	0.98	1.11	0.76	0.99
13	d/e	NGT	PD	0.91	1.12	0.93	0.75	0.69	0.96	1.35
18	no	NGT	PD	1.02	1.21	1.03	1.23	1.26	1.10	0.82
20	no	NGT	NGT	1.02	1.21	1.11	1.47	1.64	1.06	0.67
2	no	NGT	PD	1.03	1.14	1.20	1.14	1.37	0.78	0.84
3	d/e	NGT	T2DM	1.02	1.38	2.03	2.88	5.84	0.62	0.31
5	no	NGT	PD	1.12	1.17	1.59	1.12	1.78	0.48	0.80
10	no	NGT	T2DM	1.01	1.18	1.83	0.17	0.31	0.09	4.98
12	no	NGT	T2DM	1.02	1.18	1.86	8.45	16.25	1.36	0.11
14	no	PD	T2DM	1.02	1.07	2.54	2.06	5.18	0.31	0.41
1	no	IFG	NGT	1.03	1.01	0.87	1.62	1.42	1.84	0.64
4	no	IFG	T2DM	1.00	1.33	1.72	1.54	2.64	0.49	0.58
8	no	NGT	T2DM	1.01	1.40	1.02	0.67	0.68	0.74	1.46
11	no	NGT	NGT	1.01	0.96	0.97	0.64	0.63	0.79	1.55
15	d/e	NGT	T2DM	0.90	1.19	1.42	0.70	1.00	0.40	1.31
16	d/e	NGT	T2DM	1.02	2.34	3.81	0.67	2.57	0.08	0.63
17	no	IFG	IFG	0.94	0.98	0.89	0.67	0.59	0.93	1.52
19	d/e	NGT	PD	0.95	1.18	1.11	2.09	2.33	1.35	0.48

***** Numbering of persons refers to Table S6. Color code identifies persons as part of cluster 1 (red, "highly-remarkable"), cluster 2 (orange, "remarkable"), and cluster 3 (green, "unremarkable") (for details see Table S7). † Subjects received no treatment (no) or changed their diet and did physical exercises (d/e). BMI - body mass index, FPI – fasting plasma insulin, HOMA1-IR - homeostasis model assessment as an index of insulin resistance, HOMA2 %S - updated HOMA (HOMA2) of insulin sensitivity, HOMA2 %B - HOMA2 of beta cell function. Persons were categorized as NGT (normal glucose tolerance), IFG (impaired fasting glucose), PD (prediabetes), and T2DM using current diagnostic criteria of WHO and ADA for HbA_{1c} levels (≥ 6.5 % = T2DM, 5.7% $\leq HbA_{1c} \leq$ 6.4% = PD) and FPG concentrations (FPG $\geq$ 7.0 mmol/L = T2DM; 6.1 $\leq$ FPG $\leq$ 6.9 mmol/L = IFG).

Table S4. Glycated peptides targeted in tryptic digests of long-term controlled T2DM, prediabetic, and control plasma samples. The timed multiple reaction monitoring relied on the retention times in RP-HPLC and specific precursor/fragment ion pairs (Q1/Q3 mass range).

#	Sequence	Protein (Accession number, glycation site)	t_R† (min)	Q1 *m/z* (±0.2)	Q3 *m/z* (±0.2)
**1**	TCVADESAENCD**K**SLHTLFGDK	HSA (P02768; K64)	13.8	887.1	869.1
**2**	SLHTLFGD**K**LCTVATLR	HSA (P02768, K73)	17.9	698.7	680.7
**3**	ETYGEMADCCAKQEPER	HSA (P02768, K93)	8.8	746.0	136.1
**4**	ETYGEMADCCA**K**QEPER	HSA (P02768, K93)	7.9	751.3	136.1
**5**	AAFTECCQAAD**K**AACLLPK	HSA (P02768, K174)	15.9	763.0	120.0
**6**	AACLLP**K**LDELRDEGK	HSA (P02768, K181)	16.2	664.0	646.0
**7**	AEFAEVS**K**LVTDLTK	HSA (P02768, K233)	18.6	907.0	880.0
**8**	ADLA**K**YICENQDSISSK	HSA (P02768, K262)	13.6	1052.5	1025.5
**9**	TYETTLE**K**CCAAADPHECYAK	HSA (P02768, K359)	9.9	670.8	237.1
**10**	VFDEF**K**PLVEEPQNLIK	HSA (P02768, K378)	18.6	1104.1	1077.1
**11**	**K**VPQVSTPTLVEVSR	HSA (P02768, K414)	14.9	601.3	900.5
**12**	**K**QTALVELVK	HSA (P02768, K525)	13.1	645.9	603.9
**13**	EQL**K**AVMDDFAAFVEK	HSA (P02768, K545)	18.7	668.3	120.1
**14**	**K**LVAASQAALGL	HSA (P02768, K574)	16.3	652.4	501.8
**15**	VQW**K**VDNALQSGNSQESVTEQDSK	IGKC (P01834, K41)	12.7	947.1	941.1
**16**	DSTYSLSSTLTLS**K**ADYEK	IGKC (P01834, K75)	16.4	757.7	751.7
**17**	VYACEVTHQGLSSPVT**K**SFNR	IGKC (P01834, K99)	12.6	848.1	830.1
**18**	QV**K**DNENVVNEYSSELEK	FGB (P02675, K163)	12.8	762.7	72.1
**19**	**K**WDPYKQGFGNVATNTDGK	FGB (P02675, K295)	11.5	763.4	84.1
**20**	S**K**AIGYLNTGYQR	A2M (P01023, K1003)	10.5	816.9	1255.6
**21**	ALLAYAFALAGNQD**K**R	A2M (P01023, K1162)	18.5	628.7	86.1
**22**	**K**CSTSSLLEACTFR	TF (P02787, K683)	13.8	911.4	884.4
**23**	ADSSPV**K**AGVETTTPSK	IGLC (P01842, K50)	8.7	613.0	70.1
**24**	A**K**VQPYLDDFQK	APOA1 (P02647, K120)	12.5	807.4	765.4
**25**	**K**WQEEMELYR	APOA1 (P02647, K131)	10.1	530.6	524.6
**26**	AVGD**K**LPECEAVCGKPK	HP (P00738, K141)	9.4	674.0	656.0
**27**	SEET**K**ENEGFTVTAEGK	C3 (P01024, K1325)	9.2	1009.5	982.5
**28**	M**K**GLIDEVNQDFTNR	FGA (P02671, K71)	14.9	653.3	894.4
**29**	SSSYS**K**QFTSSTSYNR	FGA (P02671, K581)	8.7	664.6	658.6

†t_R - retention time. C, M, and **K** denote carbamidomethylated cysteine, methionine sulfoxide, and fructosamine lysine, respectively. HSA - human serum albumin; IGKC - Ig kappa chain c region; FGB - fibrinogen beta chain; A2M - alpha-2-macroglobulin; TF - serotransferrin; IGLC - Ig lambda chain C region; APOA1 - apolipoprotein A-I precursor; HP - haptoglobin; C3 - complement C3, FGA - fibrinogen alpha chain.

Table S5. Evaluation metrics to classify T2DM patients and controls using the quantities of 29 glycated peptides in tryptic plasma digests and glycated hemoglobin (HbA_{1c}) calculated by decision tree classifier from scikit-learn package. The optimal cut point determined for HbA_{1c} was 6.1% for all combinations.

Tested variable	Sensitivity (%)	Specificity (%)	Accuracy (%)	Cut-off
**Sequence #***				**(pmol/mg plasma protein)**
**1**	80	94	87	42.8
**2**	79	96	87	3.4
**3**	80	96	88	4.8
**4**	80	96	88	1.0
**5**	79	92	86	60.3
**6**	80	96	88	12.2
**7**	79	94	86	305.2
**8**	80	90	85	29.5
**9**	79	94	87	NA
**10**	80	94	87	51.1
**11**	79	94	87	1.1
**12**	79	92	86	2827.9
**13**	80	96	88	165.5
**14**	79	96	88	10.2
**15**	80	96	88	6.0
**16**	80	96	88	36.7
**17**	80	92	86	2.7
**18**	80	92	86	0.8
**19**	79	94	86	2.1
**20**	80	94	87	NA
**21**	80	94	87	11.4
**22**	79	92	86	3.6
**23**	79	94	86	1.3
**24**	80	96	88	1.6
**25**	81	88	84	0.3
**26**	79	92	85	0.01
**27**	79	94	86	0.7
**28**	80	96	88	0.9
**29**	80	94	87	1.0

*Numbering according to table S2. NA - a cut-off value was not available when the decision tree algorithm did not choose this feature, as its addition would decrease the classification accuracy.

Table S6: Evaluation metrics to classify T2DM patients and controls using the quantities of 29 glycated peptides in tryptic plasma digests and FPG levels calculated by decision tree classifier from scikit-learn package. The optimal cut point determined for FPG was 6.36 mmol/L for all combinations.

Tested variable	Sensitivity (%)	Specificity (%)	Accuracy (%)	Cut-off
**Sequence #***				**(pmol/mg plasma protein)**
**1**	69	86	77	35.2
**2**	71	81	76	5.3
**3**	71	86	78	8.5
**4**	71	79	75	0.3
**5**	82	84	83	60.3
**6**	73	76	74	16.4
**7**	69	89	79	231.4
**8**	77	80	79	36.3
**9**	67	82	74	5.1
**10**	78	77	77	54.8
**11**	74	77	76	1.4
**12**	75	74	74	2665.7
**13**	84	78	81	187.3
**14**	73	78	75	15.3
**15**	69	90	80	NA
**16**	69	84	76	41.0
**17**	69	86	78	NA
**18**	72	82	77	1.8
**19**	69	84	77	2.3
**20**	70	85	77	NA
**21**	69	80	74	NA
**22**	69	84	76	3.5
**23**	72	88	80	1.6
**24**	69	86	77	NA
**25**	69	79	74	NA
**26**	62	90	76	0.02
**27**	66	78	72	0.8
**28**	69	84	76	1.0
**29**	80	86	83	1.3

*Numbering according to table S2. NA - a cut-off value was not available when the decision tree algorithm did not choose this feature, as its addition would decrease the classification accuracy.

Table S7: Evaluation metrics to classify T2DM patients and controls using the quantities of 29 glycated peptides in tryptic plasma digests and C-peptide levels calculated by decision tree classifier from scikit-learn package. The optimal cut point determined for C-peptide levels was 1.52 nmol/L for all combinations.

Tested variable	Sensitivity (%)	Specificity (%)	Accuracy (%)	Cut-off
**Sequence #[*]**				**(pmol/mg plasma protein)**
**1**	76	92	84	43.1
**2**	84	92	88	6.0
**3**	71	88	79	7.3
**4**	69	80	74	1.2
**5**	88	82	85	61.2
**6**	84	88	86	18.7
**7**	80	90	85	296.1
**8**	75	82	78	46.4
**9**	86	80	83	5.1
**10**	75	84	79	64.4
**11**	76	82	79	1.7
**12**	79	90	85	2862.9
**13**	79	88	84	258.7
**14**	82	90	86	19.9
**15**	78	84	81	8.0
**16**	79	86	82	40.3
**17**	77	81	79	3.2
**18**	86	82	84	2.4
**19**	82	85	83	2.2
**20**	80	88	84	0.7
**21**	84	82	83	26.7
**22**	83	87	85	3.4
**23**	83	89	86	1.6
**24**	73	88	80	2.1
**25**	77	78	77	1.5
**26**	86	83	84	0.05
**27**	82	91	86	0.9
**28**	75	89	82	1.1
**29**	81	81	81	1.2

[*]Numbering according to table S2.

Table S8: Evaluation metrics to classify T2DM patients and controls using the quantities of 29 glycated peptides in tryptic plasma digests and FPI levels calculated by decision tree classifier from scikit-learn package. The optimal cut point determined for FPI was 148.5 pmol/L for all combinations.

Tested variable	Sensitivity (%)	Specificity (%)	Accuracy (%)	Cut-off
**Sequence #***				**(pmol/mg plasma protein)**
**1**	80	88	84	42.8
**2**	82	88	85	6.0
**3**	69	81	75	7.4
**4**	69	78	73	NA
**5**	71	81	76	79.4
**6**	80	84	82	20.7
**7**	81	88	85	296.1
**8**	76	73	74	52.4
**9**	82	83	83	6.9
**10**	86	64	75	51.9
**11**	76	82	79	1.7
**12**	79	88	84	2827.9
**13**	83	72	78	258.7
**14**	82	88	85	19.8
**15**	76	76	76	8.0
**16**	75	76	75	NA
**17**	72	82	77	3.1
**18**	77	82	79	2.4
**19**	71	79	75	2.1
**20**	71	76	74	NA
**21**	75	78	76	26.7
**22**	80	87	83	3.4
**23**	73	85	79	1.6
**24**	74	86	80	NA
**25**	73	78	75	NA
**26**	75	90	83	0.05
**27**	77	84	80	0.9
**28**	69	81	75	NA
**29**	75	81	78	NA

*Numbering according to table S2. NA – a cut-off value was not available when the decision tree algorithm did not choose this feature, as its addition would decrease the classification accuracy.

Table S9: Evaluation metrics to classify T2DM patients and controls using the quantities of 29 glycated peptides in tryptic plasma digests and homeostasis model assessment as an index of insulin resistance (HOMA1-IR) levels calculated by decision tree classifier from scikit-learn package. The optimal cut point determined for HOMA1-IR was 5.85 for all combinations.

Tested variable	Sensitivity (%)	Specificity (%)	Accuracy (%)	Cut-off
**Sequence #***				**(pmol/mg plasma protein)**
**1**	77	98	88	42.8
**2**	80	93	86	NA
**3**	80	89	84	NA
**4**	84	92	88	NA
**5**	79	88	83	NA
**6**	81	94	88	NA
**7**	77	98	88	296.1
**8**	80	92	86	NA
**9**	82	92	87	NA
**10**	78	93	85	NA
**11**	86	92	89	NA
**12**	82	96	89	2827.9
**13**	82	88	85	187.3
**14**	80	92	86	NA
**15**	80	93	86	NA
**16**	82	90	86	NA
**17**	85	92	89	NA
**18**	78	89	83	NA
**19**	79	90	84	NA
**20**	83	92	88	NA
**21**	86	92	89	NA
**22**	82	90	86	NA
**23**	83	92	87	NA
**24**	86	92	89	NA
**25**	84	92	88	NA
**26**	79	88	83	NA
**27**	84	94	89	NA
**28**	80	94	87	NA
**29**	84	94	89	NA

*Numbering according to table S2. NA – a cut-off value was not available when the decision tree algorithm did not choose this feature, as its addition would decrease the classification accuracy.

Table S10: Evaluation metrics to classify T2DM patients and controls using the quantities of 29 glycated peptides in tryptic plasma digests and updated HOMA (HOMA2) of insulin sensitivity (HOMA2 %S) levels calculated by decision tree classifier from scikit-learn package. The optimal cut point determined for HOMA2 %S was 36.6% for all combinations.

Tested variable	Sensitivity (%)	Sensitivity (%)	Accuracy (%)	Cut-off
**Sequence #[*]**				**(pmol/mg plasma protein)**
**1**	79	96	87	42.8
**2**	80	96	88	6.0
**3**	83	90	86	NA
**4**	84	88	86	NA
**5**	82	79	80	NA
**6**	77	98	87	20.7
**7**	79	96	88	296.1
**8**	79	87	83	NA
**9**	77	92	84	NA
**10**	79	86	82	54.8
**11**	79	94	87	NA
**12**	79	96	87	2827.9
**13**	92	81	86	187.3
**14**	79	98	88	20.1
**15**	81	94	87	NA
**16**	77	96	87	34.4
**17**	80	93	86	NA
**18**	77	96	87	2.4
**19**	75	89	82	NA
**20**	81	94	87	NA
**21**	84	88	86	NA
**22**	77	94	85	NA
**23**	84	90	87	NA
**24**	79	94	86	NA
**25**	76	90	83	NA
**26**	79	90	85	0.05
**27**	78	92	85	NA
**28**	78	96	87	NA
**29**	80	96	88	NA

[*] Numbering according to table S2. NA – a cut-off value was not available when the decision tree algorithm did not choose this feature, as its addition would decrease the classification accuracy.

Table S11. Spearman rank correlation coefficients (r_S) and corresponding P-values (P) of the statistical relation between each glycated peptide and different diagnostic parameters.

#*	FFA [mmol/L]		FPG [mmol/L]		HbA_{1c} [%]		HOMA1-IR		Proinsulin [pmol/L]	
	r_S	P	r_S	P	r_S	P	r_S	P	r_S	P
**1**	0.46	2.7E-06	0.62	2.0E-11	0.67	6.1E-14	0.44	6.8E-06	0.43	4.4E-05
**2**	0.39	8.1E-05	0.60	9.5E-11	0.68	3.4E-14	0.37	2.2E-04	0.37	5.2E-04
**3**	0.15	1.5E-01	0.35	4.8E-04	0.43	1.1E-05	0.28	5.0E-03	0.28	9.5E-03
**4**	0.14	1.8E-01	0.41	3.2E-05	0.44	6.1E-06	0.35	4.4E-04	0.34	1.6E-03
**5**	0.36	2.8E-04	0.67	1.4E-13	0.69	4.5E-15	0.45	3.4E-06	0.44	3.4E-05
**6**	0.41	2.8E-05	0.59	3.8E-10	0.63	4.5E-12	0.46	2.4E-06	0.43	5.0E-05
**7**	0.39	7.7E-05	0.48	5.7E-07	0.53	3.0E-08	0.37	2.5E-04	0.37	4.4E-04
**8**	0.27	7.5E-03	0.51	1.1E-07	0.60	9.7E-11	0.35	4.1E-04	0.37	5.9E-04
**9**	0.34	6.3E-04	0.67	1.4E-13	0.71	7.2E-16	0.48	6.2E-07	0.47	7.6E-06
**10**	0.35	5.0E-04	0.56	3.4E-09	0.60	1.3E-10	0.36	3.2E-04	0.38	3.9E-04
**11**	0.40	6.5E-05	0.68	3.6E-14	0.64	3.4E-12	0.49	3.3E-07	0.38	4.1E-04
**12**	0.41	3.1E-05	0.53	2.0E-08	0.65	1.2E-12	0.38	1.5E-04	0.42	7.7E-05
**13**	0.45	3.7E-06	0.59	2.5E-10	0.64	3.0E-12	0.44	6.9E-06	0.47	6.5E-06
**14**	0.37	2.2E-04	0.64	2.4E-12	0.71	5.2E-16	0.47	1.3E-06	0.44	2.7E-05
**15**	0.16	1.1E-01	0.45	3.7E-06	0.56	2.4E-09	0.29	3.8E-03	0.24	2.6E-02
**16**	0.13	2.1E-01	0.40	5.4E-05	0.52	4.5E-08	0.24	1.7E-02	0.19	8.4E-02
**17**	0.12	2.4E-01	0.35	5.0E-04	0.42	2.6E-05	0.19	5.9E-02	0.12	2.6E-01
**18**	0.43	1.3E-05	0.67	1.1E-13	0.76	1.7E-19	0.58	8.6E-10	0.54	9.1E-08
**19**	0.32	1.4E-03	0.54	9.4E-09	0.67	1.2E-13	0.46	3.0E-06	0.42	7.7E-05
**20**	0.28	5.6E-03	0.43	1.1E-05	0.52	5.3E-08	0.39	9.4E-05	0.33	2.4E-03
**21**	0.36	3.4E-04	0.48	8.9E-07	0.51	1.4E-07	0.39	6.9E-05	0.37	6.0E-04
**22**	0.27	8.8E-03	0.60	1.4E-10	0.64	1.7E-12	0.43	1.2E-05	0.40	1.6E-04
**23**	0.26	1.2E-02	0.58	5.6E-10	0.61	4.5E-11	0.47	1.2E-06	0.41	1.1E-04
**24**	0.15	1.4E-01	0.49	4.6E-07	0.54	1.3E-08	0.30	3.0E-03	0.23	3.1E-02
**25**	0.09	3.8E-01	0.29	4.1E-03	0.31	2.2E-03	0.14	1.6E-01	0.01	9.3E-01
**26**	0.35	4.0E-04	0.64	3.4E-12	0.65	1.1E-12	0.62	2.6E-11	0.53	2.4E-07
**27**	0.34	6.7E-04	0.70	2.9E-15	0.68	1.4E-14	0.54	1.8E-08	0.52	3.7E-07
**28**	0.22	2.9E-02	0.36	3.2E-04	0.50	1.8E-07	0.37	1.8E-04	0.35	1.3E-03
**29**	0.30	2.6E-03	0.57	1.4E-09	0.70	2.6E-15	0.53	2.8E-08	0.50	1.2E-06

*Sequence numbering corresponds to peptides listed in Table 2. FFA - free fatty acids, FPG - fasting plasma glucose, HbA_{1c} - glycated hemoglobin, HOMA1-IR - homeostasis model assessment as an index of insulin resistance.

Table S12: Receiver operating characteristic (ROC) analysis. ROC parameters were estimated for peptide levels of all 29 glycated peptides quantified in tryptic digests of plasma samples obtained from 48 long-term controlled T2DM patients and 48 controls. For comparison, ROC parameters of HbA_{1C} and fasting plasma glucose (FPG) are listed.

Tested variable	AUC[†] (%)	Sensitivity (%)	Specificity (%)	Cut-off
**Sequence #[*]**				**(pmol/mg plasma protein)**
**1**	81	75	77	33.1
**2**	78	75	71	4.2
**3**	62	52	73	4.7
**4**	60	62	60	0.7
**5**	78	67	85	55.8
**6**	76	79	60	12.6
**7**	76	73	67	229.0
**8**	71	60	85	36.5
**9**	77	67	81	4.8
**10**	78	67	81	52.1
**11**	76	69	79	1.4
**12**	77	75	73	2,280.0
**13**	82	75	75	179.0
**14**	76	73	81	13.1
**15**	67	56	69	6.1
**16**	65	52	81	38.1
**17**	64	50	83	2.9
**18**	79	69	88	1.8
**19**	74	60	81	2.2
**20**	67	52	85	0.6
**21**	71	60	79	21.1
**22**	72	60	79	3.0
**23**	74	56	85	1.4
**24**	64	58	67	1.6
**25**	57	44	75	1.3
**26**	77	67	85	0.03
**27**	76	67	75	0.7
**28**	66	50	83	1.0
**29**	73	58	83	1.1
				**(%)**
**HbA_{1c}**	89	63	98	6.5
	89	81	96	6.0
				**(mmol/L)**
**FPG**	81	54	98	7.00
	81	79	83	5.72

[*]Sequence numbers correspond to peptides listed in Table S2. [†]AUC - Area under the curve.

Table S13. Cut-off values of glycated peptides used for subgrouping prediabetic patients.

#*	Cut-off [pmol/mg plasma protein]	Number of samples above the cut-off
**1**	35.50	5
**2**	7.00	4
**3**	5.10	9
**4**	2.10	4
**5**	65.00	8
**6**	14.40	9
**7**	231.00	5
**8**	28.00	12
**9**	6.00	7
**10**	64.00	8
**11**	1.47	8
**12**	2530.00	12
**13**	155.00	12
**14**	16.10	7
**15**	6.00	5
**16**	39.50	-
**17**	2.50	11
**18**	1.60	3
**19**	2.20	below LOD♣
**20**	0.70	10
**21**	16.9	7
**22**	4.00	6
**23**	1.32	10
**24**	1.27	9
**25**	1.86	6
**26**	0.029	7
**27**	0.70	0
**28**	0.84	3
**29**	1.05	6

*Numbering according to table S2.♣Glycation degrees of all 20 subjects where below the LOD.

Table S14. Affiliation of prediabetic persons to clusters based on cluster analysis or manual counting.

Subject #	Number of glycation sites above the cut-off]	Affiliation to cluster # (done by cluster analysis)[a]	Affiliation to cluster # (done by manual counting)[b]
**1**	3	3	3
**2**	6	2	2
**3**	10	2	2
**4**	0	3	3
**5**	11	2	2
**6**	18	2	1
**7**	22	1	1
**8**	2	3	3
**9**	21	1	1
**10**	12	2	2
**11**	2	3	3
**12**	6	2	2
**13**	21	1	1
**14**	11	2	2
**15**	0	3	3
**16**	1	3	3
**17**	2	3	3
**18**	24	1	1
**19**	2	3	3
**20**	19	1	1

[a] Cluster number was assigned by cluster analysis (see figure S1). [b] Clustering considering the number of glycated peptide quantities above the cut-off values (cluster 1: 18-24 counts ≡ “highly-remarkable”, cluster 2: 6-12 counts ≡ “remarkable”, cluster 3: 0-3 counts ≡ “unremarkable”).

Table S15. Ranking of features for the classification of 20 prediabetic subjects based on random forest feature importance.

#	Abbreviation glycation site*	Feature importance*
**1**	HSA K262	0.032362
**2**	HSA K378	0.024121
**3**	HSA K73	0.022837
**4**	HSA K525	0.01774
**5**	HSA K574	0.009985
**6**	HSA K359	0.006306
**7**	TF K 683	0.005847
**8**	HSA K174	0.004004
**9**	HSA K64	0.003771

*Abbreviations according to table S2. Feature importance is calculated using Scikit-Learn. The square of their values is shown.

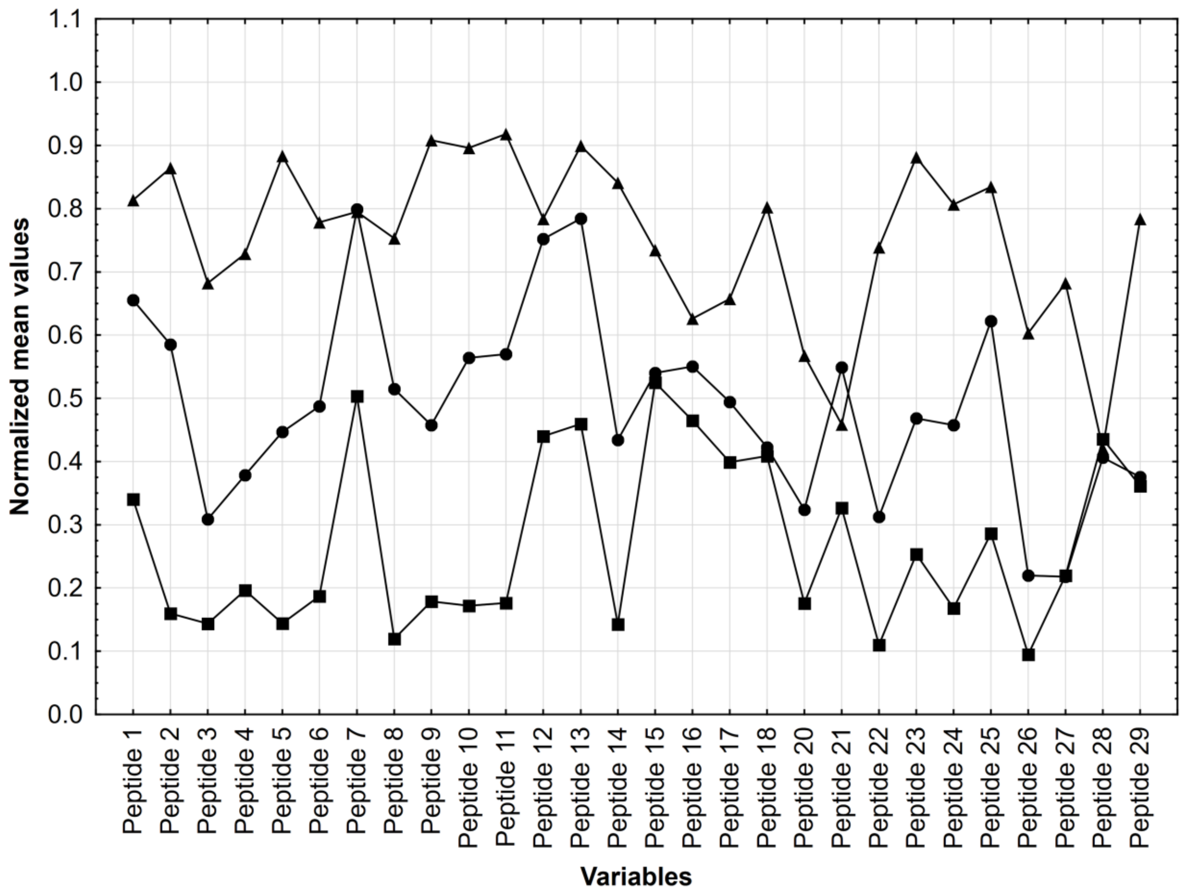

Figure S1. Graph of continuous variable means of a K-Means cluster analysis for 20 prediabetic subjects. The plotted values of cluster 1 (triangle), cluster 2 (circle), and cluster 3 (square) depict the means scaled to the overall ranges of observed values for the respective continuous variables (peptide glycation degree).

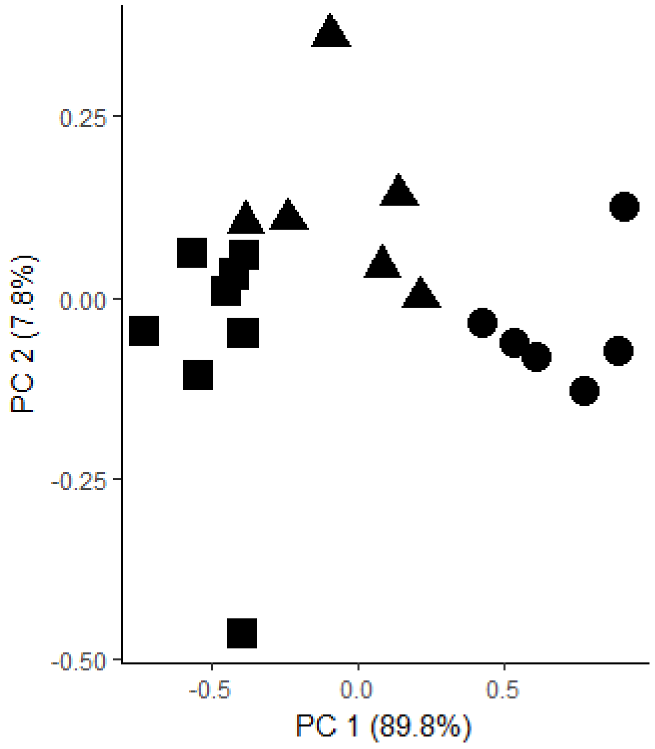

Figure S2. Principle component plot of 20 prediabetic subdivided into three clusters (circle = highly remarkable, triangle = remarkable, square = unremarkable) using nine glycated peptides including eight glycation sites of HSA (Lys262, Lys378, Lys73, Lys525, Lys574, Lys359, Lys174, Lys64) and one of serotransferrin (Lys683). PC1 and PC2 denote first and second principle components, respectively.
